# Case report: discovery of 2 gene variants for aromatic L-amino acid decarboxylase deficiency in 2 African American siblings

**DOI:** 10.1186/s12883-019-1596-8

**Published:** 2020-01-09

**Authors:** Berrin Monteleone, Keith Hyland

**Affiliations:** 1 NYU Langone Health Winthrop Pediatric Associates, 120 Mineola Blvd., Suite 210, Mineola, NY 11501 USA; 2 LabCorp, Atlanta, GA USA

**Keywords:** Rare disease, Genetic therapy, Neurotransmitters, Dopamine

## Abstract

**Background:**

Aromatic l-amino acid decarboxylase (AADC) deficiency is a rare genetic disorder with heterogeneous phenotypic spectrum resulting from disease-causing variants in the dopa decarboxylase (*DDC*) gene. Consensus guidelines recommend dopamine agonists, monoamine oxidase inhibitors, and other symptomatic treatments, but most patients have an unrelenting disease course with no response to these therapies.

**Case presentation:**

We describe 2 African American siblings with AADC deficiency and identify 2 *DDC* gene variants not previously associated with the disorder. The patients were evaluated for cognitive and neurologic impairments. Diagnosis of AADC deficiency was initially based on evaluation of urine and plasma metabolites, followed by targeted *DDC* gene sequencing. The first patient, a firstborn African American female, had moderate elevations of vanillactic and vanilpyruvic acids, and slight elevation of N-acetylvanilalanine in urine. The second patient, an African American female and younger sibling of the first patient, had low AADC enzyme activity and elevated 3-*O*-methyldopa levels in plasma. Genetic testing confirmed that both siblings possessed the same 2 *DDC* gene variants, which were identified as NM_000790.3: c.48C > A (p.Tyr16Ter) and NM_000790.3: c.116G > C (p.Arg39Pro).

**Conclusions:**

This report describes 2 previously unknown patients with AADC deficiency and confirmed the presence of 2 *DDC* gene variants not previously associated with this disorder. Further research is needed to identify disease-modifying treatments for this devastating neurometabolic disorder. Gene therapy with a recombinant adeno-associated viral vector serotype 2 carrying the gene for the human AADC protein (AAV2-hAADC) is currently in clinical development.

## Background

Aromatic l-amino acid decarboxylase (AADC) deficiency is a rare neurometabolic disorder with heterogeneous phenotypic spectrum resulting from disease-causing variants of the dihydroxyphenylalanine (l-DOPA) decarboxylase (*DDC*) gene [1–4]. The AADC enzyme is required for the final decarboxylation step that results in synthesis of monoamine neurotransmitters, most importantly dopamine [5]. Decreased activity of the AADC enzyme results in severe dopamine deficits, is directly linked to a wide range of neurologic and cognitive impairments [5], and ultimately results in the failure of patients with AADC deficiency to achieve developmental motor milestones [3].

Little is known at this time about global prevalence of AADC deficiency. Taiwan is the site of a potential founder mutation (IVS6 + 4A > T), and the incidence of AADC deficiency in Taiwan has been estimated at 1/32,000 births [6]. A recent analysis of the estimated prevalence of AADC deficiency in the United States, European Union, and Japan demonstrated conservative population prevalence (2016 data) of 840, 853, and 125, respectively [7]. In a separate evaluation of disease prevalence, 36 new patients with AADC deficiency were identified via screening of biological samples obtained between 2008 and 2016 from 19,684 US patients with neurologic deficits of unknown origin [8].

In general, patients with AADC deficiency exhibit arrested motor development with no full head control and no ability to sit, stand, or walk [9]. Consensus guidelines specifically describe clinical features associated with the central and autonomic nervous systems (CNS, ANS) [3, 10]. Key symptoms associated with CNS dysfunction include truncal hypotonia, poor head control, limb hypertonia, dyskinesia, dystonia, oculogyric crisis, hypokinesia and/or bradykinesia, delayed developmental milestones (motor, cognitive, and speech), irritability, dysphoria, and insomnia. Clinical features associated with ANS dysfunction include ptosis, miosis, profuse drooling, postural hypotension, excessive sweating, temperature instability, nasal congestion, impairment of sympathetic blood pressure modulation, feeding/swallowing problems, gastroesophageal reflux, constipation, and diarrhea [3, 10, 11]. Importantly, many of the neurological features associated with AADC deficiency may be observed in seizure disorders and cerebral palsy, which may lead to an incorrect diagnosis of these more commonly occurring conditions [12–14]. AADC deficiency is typically diagnosed through analysis of neurotransmitter metabolite levels in cerebrospinal fluid (CSF) according to consensus guidelines [15]. Because clinical presentation typically begins soon after birth [16], efforts have been made to standardize analysis of dried bloodspots for routine screening of AADC deficiency in newborns [6]. Currently, limited treatment options are available for individuals diagnosed with AADC deficiency and none are curative. Patients require lifelong care, and premature death typically occurs prior to the age of 10 years [17]. However, gene therapies are currently being developed clinically as potential novel treatment options for these patients.

The purpose of this case report is to describe the clinical presentation and diagnosis of 2 previously unknown patients with AADC deficiency.

## Case presentation

### Case 1

The first proband is a female, the firstborn child of a 19-year-old mother at 38 weeks’ gestation via vaginal vertex delivery. She is the daughter of African American, nonconsanguineous parents. The mother had a normal anatomic scan of the fetus, and prenatal screening was positive for the mother being a carrier of sickle cell anemia and of spinal muscular atrophy (SMA). The patient tested negative, with the presence of 2 copies of the survival motor neuron (SMN1) gene. Growth parameters at birth are detailed in Table 1.

**Table 1:** Growth Parameters, Exemplary Case #1

Parameter	At Birth	Percentile at Birth	3 Years of Age	Percentile at 3 Years of Age
Weight, kg	3.6	78	12.9	24.95
Length, cm	53	98	92	24.02
Head circumference, cm	N/A	N/A	47	15

*N/A* not available

The parents first noticed developmental delay at 6 months of age. The child did not transfer objects at 18 months and could not roll over at 3 years. While she started sitting with support at 12 months, at 3 years of age she is still unable to sit alone. At the age of 3 years, she can pull herself to standing with someone supporting her from behind, but she is still unable to crawl or walk with help. She said her first words at age 12 months and was able to use some 2-word phrases. Her mom was able to understand her limited speech, and the patient was able to follow simple commands. Feeding issues began to arise, and she now gags on some solid foods. Growth parameters at 3 years of age can be found in Table 1.

At the age of 7 months she was seen by a neurologist, initially for hypotonia, developmental delays, and intermittent episodes of decreased tone and eye crossing. During these episodes she was usually tired but continued to be interactive. She had to sleep 30 min to an hour to come out of a spell, and when she woke up she was back to her baseline. Her spells lasted for hours at a time, but there was never any loss of consciousness.

On physical exam, she had oculogyric crisis, dystonia, and hypokinesia. Otherwise, she had a normal physical and vision exam.

There have been no concerns with her sleep. She has a history of some nasal congestion as an infant. She also has a history of some autonomic symptoms, such as abnormal sweating.

At the age of 17 months, magnetic resonance imaging (MRI) of her brain demonstrated a subtle area of chronic tissue loss in the subependymal white matter in the posterior left frontal area. The lateral and third ventricles were borderline in size, indicating mild ventriculomegaly. Seizure disorders were considered due to abnormal eye movements, but multiple electroencephalograms (EEGs) showed no seizure activity. However, there was abnormal activity that was suggestive of a metabolic and structural etiology.

Plasma amino acids and total and free carnitine levels were within normal limits. Urine organic acids revealed moderate elevations of vanillactic and vanilpyruvic acids with slight elevation of N-acetylvanilalanine, a pattern suggestive of AADC deficiency [18].

Next-generation sequencing of the proband revealed 2 variants in the *DDC* gene: NM_000790.3: c.48C > A (p.Tyr16Ter) and NM_000790.3: c.116G > C (p.Arg39Pro). Analysis of amino acid conservation indicates that the wild-type amino acid, Arg39, is completely conserved in all 98 vertebrates examined, increasing the likelihood that a change at this position would not be tolerated and could adversely affect the function of the protein. As far as we are aware, these variants have not been previously reported in association with AADC deficiency. Sanger sequencing confirmed the results. Additional evidence for pathogenicity was provided by a finding of low AADC activity (2.13 pmol/min/mL [normal range = 36–129 pmol/min/mL]) and elevated 3-OMD (3733.0 nmol/L [normal range = 64–280 nmol/L]) in plasma.

A chromosome microarray revealed a 677 kb copy gain in chromosome 4q27 (122,570,932 – 123,248,135) that contained OMIM genes ANXA5, EXOSC9, CCNA2, BBS7, TRPC3, and KIAA1109 as well as the genes TMEM155 and PP12613 with unknown clinical significance. However, fluorescence in situ hybridization analysis using an oligonucleotide clone (RP11-184P16) from the duplicated region was inconclusive and could not confirm the microarray result in the metaphase analysis. The interphase analysis identified two RP11-184P16 signals in 86% of the nuclei.

Following diagnosis of AADC deficiency, the proband started receiving vitamin B6 with no clinical improvement. She also initiated physical, speech, feeding, and occupational therapies.

### Case 2

Case 2 is the female sibling of the proband. She was born at 41 weeks via normal spontaneous vaginal delivery. Growth parameters at birth are depicted in Table 2. The pregnancy was uncomplicated. Anatomic scan was normal. A cell-free DNA analysis was normal as well. Her parents noticed abnormal eye and arm movements in the first months of life. She was able to smile at 2 weeks and roll over at 4 months. At 6 months, she was unable to sit alone or hold her bottle. At this time, both a brain MRI and an EEG were normal. At 8 months, she reached for objects.

**Table 2:** Growth Parameters, Exemplary Case #2

Parameter	At Birth	Percentile at Birth	12 Months of Age	Percentile at 12 Months of Age
Weight, kg	3.6	78	7.25	4.18
Length, cm	53	98	69.60	4.63
Head circumference, cm	N/A	N/A	45	53.68

*N/A* not available

At 12 months, she was not able to sit alone or transfer objects from one hand to the other. She was able to sit with support but could not crawl or pull herself up. At this time, she had hypotonia, developmental delay, oculogyric crisis, dystonia, and hypokinesia. She did not have any sleep difficulties. Targeted genetic testing revealed the same 2 variants in the *DDC* gene that were detected in her older sister (Case 1). In plasma analysis, AADC activity was low at 2.36 pmol/min/mL (normal range = 36–129 pmol/min/mL) and 3-OMD was elevated at > 5000 nmol/L (normal range = 64–280 nmol/L), confirming the diagnosis of AADC deficiency. A chromosome microarray demonstrated normal female, 46, XX.

The patient has been receiving vitamin B6 since her diagnosis of AADC deficiency with no clinical improvement and has initiated physical, speech, feeding, and occupational therapies.

## Discussion and Conclusion

AADC deficiency is a rare and life-threatening neurometabolic disorder with no currently available curative treatment options. It is commonly misdiagnosed, and little is known at this time about its global prevalence. In addition to observation of the previously described clinical symptoms, there are currently 3 main methodologies used to diagnose AADC deficiency [3]. The first involves the measurement of serotonin and catecholamine metabolites in CSF. Homovanillic acid (HVA), 5-hydroxyindoleacetic acid (5-HIAA), and 3-methoxy-4-hydroxyphenylglycol (MHPG) are drastically reduced in the CSF of patients with AADC deficiency. In addition, their precursors, 3-*O*-methyldopa (3-OMD), l-DOPA, and 5-hydroxytryptophan (5-HTP), are typically increased [3]. The second methodology involves the measurement of AADC enzyme activity in plasma [18]. A marked decrease in AADC plasma activity has been observed in all AADC cases where it has been measured [3]. A third diagnostic measure is genetic testing. There are currently 82 *DDC* variants listed in the Pediatric Neurotransmitter Disease database (PNDdb; available at: http://biopku.org/pnddb/home.asp), including 79 variants that may be associated with the disease phenotype [19]. Current guidelines recommend that genetic testing be performed to diagnose AADC deficiency. To confirm diagnosis, 2 of the 3 previously described diagnostic measurements should be positive [3]. Additional diagnostic methodologies for AADC deficiency include measurement of 3-OMD in blood or dried blood spots. Levels of 3-OMD have been shown to be highly elevated in dried blood spots from newborns with AADC deficiency, and this methodology has potential as a newborn screening tool [6]. In addition, elevated urine levels of vanillactic and vanilpyruvic acids and N-acetylvanilalanine may be suggestive of AADC deficiency [20].

In this case report, we described 2 previously unknown patients with AADC deficiency: 2 siblings with 2 new *DDC* gene variants. Both siblings presented with developmental deficiencies within the first few months of life. In the first reported case, elevations in urine vanillactic acid, vanilpyruvic acid, and N-acetylvanilalanine levels suggested AADC deficiency and were confirmed by genetic sequencing of the *DDC* gene. Further testing confirmed reduced AADC activity. In the second reported case, an initial diagnosis was made at an earlier age due to the older sister’s diagnosis and was based on *DDC* gene sequencing, confirmed by plasma analysis of AADC activity.

While a number of management strategies, treatments, and therapeutic modalities have been aimed at ameliorating the effects of AADC deficiency, they have all been based on small populations and have shown widely varying degrees of success. Current consensus guidelines suggest selective dopamine agonists, monoamine oxidase (MAO) inhibitors, and PLP/pyridoxine (i.e., vitamin B6) as first-line treatment agents. Symptomatic treatment agents include anticholinergic agents, melatonin, benzodiazepines, and alpha-adrenoceptor blockers. The use of multiple concurrent treatments is needed in most cases, but even with multiple treatments the management of symptoms is often ineffective [3]. There are currently no curative treatments available for AADC deficiency; the long-term benefits of symptomatic treatments in patients with AADC deficiency in terms of quality of life and prognosis remain unclear. There is also a dearth of clinical trials investigating treatment of this rare disease.

A recombinant adeno-associated viral vector serotype 2 carrying the gene for the human AADC protein (AAV2-hAADC) has recently been tested in children with AADC deficiency. Results have been promising, with improvements seen in cognitive abilities, neuromotor abilities, and body weight [21].

Gene therapy appears to be a viable treatment option with promising results. More research is needed to create a comprehensive gene therapy approach to the treatment of AADC deficiency [21]. The parents of the 2 patients described in this case report are appreciative of the treatment their children have received and have also requested to be involved in any kind of treatment-related research that could potentially benefit their children, demonstrating the support among families affected by AADC deficiency for improved treatment options.

In this case report, we described the clinical presentation and diagnosis of 2 previously unknown patients with the rare neurometabolic disorder of AADC deficiency and confirmed the presence of 2 *DDC* gene variants not previously associated with this disorder. Both patients presented with developmental deficiencies within the first few months of life, and diagnoses of AADC deficiency were made based on neurotransmitter metabolite levels, genetic sequencing and AADC enzyme activity analyses. Traditional treatment approaches have been focused on dopamine agonists, MAO inhibitors, and PLP/pyridoxine. Gene therapy has recently been introduced as a new and promising therapeutic modality. Further education, research, and discussion are needed to ensure proper and timely diagnosis of AADC deficiency.

## Data Availability

Data sharing not applicable to this article as no datasets were generated or analyzed during the current study.

## Abbreviations

3-OMD: 3-*O*-methyldopa
5-HIAA: 5-hydroxyindoleacetic acid
5-HTP: 5-hydroxytryptophan
AADC: Aromatic l-amino acid decarboxylase
AAV2-hAADC: Adeno-associated viral vector serotype 2 carrying the gene for the human AADC protein
ANS: Autonomic nervous system
CNS: Central nervous system
CSF: Cerebrospinal fluid
DDC: Dopa decarboxylase
EEG: Electroencephalogram
HVA: Homovanillic acid
l-DOPA: Dihydroxyphenylalanine
MAO: Monoamine oxidase
MHPG: 3-methoxy-4-hydroxyphenylglycol
MRI: Magnetic resonance imaging
PNDdb: Pediatric Neurotransmitter Disease database
SMA: Spinal muscular atrophy
SMN1: Survival motor neuron
